# Safety evaluation of early drain removal following pancreaticoduodenectomy: A single-center retrospective cohort study

**DOI:** 10.3389/fonc.2022.993901

**Published:** 2022-09-29

**Authors:** Xuehai Xie, Kai Chen, Zonghao Liu, Feng Wang, Yongsu Ma, Shupeng Zhang, Zhijiang Shao, Yinmo Yang, Xiaodong Tian

**Affiliations:** ^1^ Department of General Surgery, Peking University First Hospital, Beijing, China; ^2^ Department of Endoscopy Center, Peking University First Hospital, Beijing, China; ^3^ Department of General Surgery, Tianjin Fifth Centre Hospital, Tianjin, China

**Keywords:** pancreaticoduodenectomy, early drain removal, postoperative pancreatic fistula, postoperative complications, pancreatic cancer

## Abstract

**Objectives:**

The effects of early drain removal (EDR) on postoperative complications after pancreaticoduodenectomy (PD) remains to be investigated. This single-center retrospective cohort study was designed to explore the safety of EDR after PD.

**Methods:**

A total of 112 patients undergoing PD with drain fluid amylase (DFA) on postoperative day (POD) 1 and 3 <= 5000 were divided into EDR and late drain removal (LDR). Propensity Score Matching (PSM) was used. We compared postoperative outcomes between two groups and explore the risk factors of total complications using univariate and multiple logistic regression analyses.

**Results:**

No statistical differences were found in primary outcomes, including Grade B/C postoperative pancreatic fistula (POPF) (Original cohort: 5.71% vs. 3.90%; P = 1.000; PSM cohort: 3.33% vs. 6.67%; P = 1.000), and total complications (Original cohort: 17.14% vs. 32.47%; P = 0.093; PSM cohort: 13.33% vs. 33.33%; P = 0.067). The EDR was associated with shorter in-hospital stay (Original cohort: 11 days vs. 15 days; P < 0.0001; PSM cohort: 11 days vs. 15 days; P < 0.0001).

**Conclusions:**

EDR on POD 3 is safe for patients undergoing PD with low risk of POPF.

## Introduction

With the rapid development of surgical technique in the last decades, the perioperative mortality of pancreaticoduodenectomy (PD) decreased significantly, whereas the incidences of postoperative complications are still high (1–3). The postoperative pancreatic fistula (POPF) remains one of the most significant postoperative complications after PD, which significantly increases postoperative in-hospital stay and medical burden (4). A growing body of study proposed the predicted models to evaluate the risk of POPF after pancreatic surgery (5, 6). The management of intraperitoneal drainage plays a crucial role in the process of postoperative recovery of patients undergoing PD. The detection of drain fluid around the operative area is perceived as an important indicator for early identifying POPF, postpancreatectomy hemorrhage (PPH) or intra-abdominal infection, therefore prophylactic drainage placement during PD is accepted in most of pancreatic centers (7). However, many studies raised concerns about the placement of intraperitoneal drainage after PD. For example, Conlon et al. (8) performed the first randomized controlled trial (RCT) to demonstrate that the placement of drainage after pancreatic resection failed to reduce postoperative complications, but increased the incidences of intra-abdominal collections and infection. Subsequently, multiple RCTs and meta-analysis proved the safety of omission of drainage after pancreatic resection (9–13). However, one RCT was stopped because of the significantly increased mortality from 3% to 12% for patients undergoing PD without the placement of intraperitoneal drainage (14). Therefore, no consensus was reached with regard to whether to place prophylactic intraperitoneal drainage.

Recently, multiple studies paid more attention to evaluating the feasibility of EDR. Bassi et al. (15) performed the first RCT to explore the safety of EDR, and results showed that EDR significantly decreased complications, in-hospital stay and costs than late drain removal (LDR). Thereafter, Dai et al. (16, 17) performed single and multiple-center RCT to compare EDR and LDR regarding Clavien-Dindo grades 2–4 complications. The strict inclusion criteria were used to select patients with low or middle risk of POPF, which demonstrated EDR is safe in selected patients. The American College of Surgeons’ National Surgical Quality Improvement Program (ACS-NSQIP) was also utilized to explore the effects of EDR on postoperative complications for PD. EDR after PD was associated with better outcomes (18). Although the safety of EDR after PD was proved preliminarily, the low risk patient selection criteria and the time-point of EDR remains to be further explored.

Here, we designed single-center retrospective cohort study to the confirm the safety of EDR on POD 3 for PD patients with the low risk of POPF. A total of 112 patients undergoing PD with drain fluid amylase (DFA) on POD 1 and 3 <= 5000 were divided into EDR and LDR groups. Propensity Score Matching (PSM) was used. We found that there were no significant differences in Grade B/C POPF and total complications. In addition, EDR was associated with shorter in-hospital stay.

## Methods

### Single-center study design

This retrospective cohort study was approved by the Ethical Committee on Peking University First Hospital (Approval No.2021-636) and performed in accordance with the Helsinki Declaration. The consecutive patients undergoing pancreaticoduodenectomy (PD) or pylorus preserving PD (PPPD) from January 2017 and December 2020 in our institution with drain fluid amylase (DFA) on both postoperative day (POD) 1 and 3 ≤ 5000 U/L were enrolled. Specific exclusion criteria consisted in (a) patients underwent distal pancreatectomy (DP) or total pancreatectomy; (b) DFA on POD 1 or 3 > 5000 U/L; (c) patients with age < 18; (d) incomplete records of key postoperative outcomes.

The time-point of early and late drain removal was defined as POD 3 and ≥ POD 5. All the operations were performed by experienced pancreatic surgeons at our institution. Clinicopathological data were collected retrospectively through electronic medical record system.

### The primary and secondary outcomes in single-center study

Postoperative complications were evaluated using the Clavien–Dindo classification system (19). The postoperative complications such as postoperative pancreatic fistula (POPF) (20), delayed gastric emptying (DGE) (21), and postpancreatectomy hemorrhage (PPH) (22) were in accordance with the consensus definition of the International Study Group of Pancreatic Fistula (ISGPF). Intra-abdominal collections were defined as collection of fluid measuring at least 3 cm in diameter demonstrated by ultrasound or CT scan. The primary outcomes in this study included Grade B/C POPF and total complications. The secondary outcomes were DGE, PPH, intra-abdominal collections, wound infection, re-operation, re-admission and post-operative in-hospital stay.

### Propensity score matching

Propensity Score Matching was used to deal with confounding factors using R package “MatchIt”. Matching variables included age, BMI, pancreatitis, diabetes, cardiovascular disease, soft pancreatic texture, operation time, blood loss, diameter of main pancreatic duct, PPPD, vascular resection, ASA scores, pathology. The nearest neighbor matching method with a tolerate of 0.1 was selected.

### Statistical analysis

Data was summarized as mean ± standard deviation or median (interquartile range, IQR) for continuous variables subjected to normal distribution or no normal distribution. The independent-samples t test and Mann-Whitney U test was performed to compare continuous variables between two groups. For categorical variables, data was summarized as frequency (ratio) and the chi-square test, fisher exact test, or rank sum test was used. Study of potential prognostic factors for total complications was carried out using univariate and multiple logistic regression analyses. All statistical analyses were conducted using SPSS version 22.0 software (SPSS22, Chicago, USA). Statistical significance was defined as p < 0.05.

## Results

### Characteristics of patients in single-center study

A total of 112 patients who underwent PD performed at our institution between January 2017 and December 2020 were divided into two groups: EDR (n = 35, drains were removed on POD 3) and LDR (n = 77, drains were removed on or beyond POD 5). Patients with amylase value in drains on POD 1 or 3 > 5000 U/L were excluded. The patients previously enrolled in the multi-center study performed by Peking Union Medical College Hospital were not included in our cohort.

The demographic, surgical, biochemical, and pathological characteristics of patients were summarized in **Table 1**. There were no significant differences in gender, BMI, pancreatitis, diabetes, cardiovascular disease, smoke, alcohol, intraoperative RBC transfusion, PPPD, vascular resection, preoperative hemoglobin, serum total bilirubin, and pathology between two groups. No significant differences were found as well with particular regard to risk factors of POPF (soft pancreatic texture, blood loss, diameter of main pancreatic duct, DFA on POD 1/3). Only two patients underwent neoadjuvant chemotherapy (2 in EDR, 0 in LDR, P = 0.847). Patients in EDR had lower age (60.23 ± 10.66 vs. 65.32 ± 12.55; P = 0.04), shorter operation time (266 [240 – 300] vs. 301 [251.5 - 418]; P = 0.026), and different ASA scores (Grade I: 17.14% vs. Grade I: 2.60%; P = 0.035) in comparison with LDR. In order to reduce the impact of confounding factors to make two groups more homogeneous, Propensity Score Matching (PSM) was conducted. After PSM, all demographic, surgical, biochemical characteristics, and risk factors of POPF were similar without significant differences between these two groups (**Table 1**). In addition, the drain placement time was 3 days in EDR group versus 11 days (3 - 15.75) in LDR group (**Table 2**).

**Table 1:** Demographic characteristics of enrolled participants in retrospective cohort study.

	Original Cohort				Propensity Score Matching			
	Total(n = 112)	EDR(n = 35)	LDR(n = 77)	P_Value	Total(n = 60)	EDR(n = 30)	LDR(n = 30)	P_Value
Age (year, mean ± SD)	63.73 ± 12.18	60.23 ± 10.66	65.32 ± 12.55	0.040*	60.78 ± 11.35	60.63 ± 11.32	60.93 ± 11.57	0.919
Gender [female, n (%)]	47 (41.96)	12 (34.26)	35 (45.45)	0.267	23 (38.33)	10 (33.33)	13 (43.33)	0.426
BMI (kg/m2, mean ± SD)	23.60 ± 3.22	23.31 ± 2.82	23.74 ± 3.40	0.517	23.13 ± 2.95	23.02 ± 2.43	23.24 ± 3.43	0.773
Pancreatitis [n (%)]	17 (15.18)	7 (20.00)	10 (12.99)	0.338	11 (18.33)	7 (23.33)	4 (13.33)	0.317
Diabetes [n (%)]	31 (27.68)	13 (37.14)	18 (23.38)	0.131	20 (33.33)	9 (30.00)	11 (36.67)	0.584
Cardiovascular Disease [n (%)]	19 (16.96)	5 (14.29)	14 (18.18)	0.611	10 (16.67)	5 (16.67)	5 (16.67)	1.000
Smoke [n (%)]	36 (32.14)	11 (31.43)	25 (32.47)	0.913	19 (31.67)	8 (26.67)	11 (36.67)	0.405
Alcohol [n (%)]	25 (22.32)	6 (17.14)	19 (24.68)	0.375	14 (23.33)	6 (20.00)	8 (26.67)	0.542
Neoadjuvant Chemotherapy [n (%)]	2 (1.79)	0 (0)	2 (2.60)	0.847	1 (1.67)	0 (0)	1 (3.33)	1.000
Soft Pancreatic Texture [n (%)]	42 (37.50)	13 (37.14)	29 (37.66)	0.958	24 (40.00)	12 (40.00)	12 (40.00)	1.000
Operation Time (min, IQR)	293.5 (244 - 360)	266 (240 - 300)	301 (251.5 - 418)	0.026*	265.5 (240 - 313)	265.5 (240 - 300)	268 (238.25 - 328.50)	0.684
Blood Loss (ml, IQR)	200 (100 - 300)	200 (100 -400)	200 (100 -300)	0.856	200 (100 - 300)	200 (100 - 400)	180 (100 - 300)	0.560
Intraoperative RBC transfusion [n (%)]	17 (15.18)	5 (14.29)	12 (15.58)	0.859	10 (16.67)	5 (16.67)	5 (16.67)	1.000
Diameter of Main Pancreatic Duct < 3 mm [n (%)]	50 (44.64)	12 (34.29)	38 (49.35)	0.137	20 (33.33)	10 (33.33)	10 (33.33)	1.000
PPPD [n (%)]	73 (65.18)	25 (71.43)	48 (62.34)	0.349	44 (73.33)	23 (76.67)	21 (70.00)	0.559
DFA on POD 1 (U/L, IQR)	470.5 (117.25 - 1722)	256 (128 -1532)	618 (114.5 - 1797.5)	0.292	606.5 (137 - 1786)	256.5 (101.5 - 1579.5)	941.5 (203.75 - 2733)	0.056
DFA on POD 3 (U/L, IQR)	175.5 (30 - 799.75)	115 (20 - 861)	177 (32 - 789.5)	0.778	291.5 (35 - 1023.25)	229.5 (26 - 1100.5)	499.5 (84.25 - 1050.5)	0.255
Vascular Resection [n (%)]	4 (3.57)	3 (8.57)	1 (1.30)	0.170	0 (0)	0 (0)	0 (0)	NA
ASA Score [n (%)]								
Grade I	8 (7.14)	6 (17.14)	2 (2.60)	0.035*	6 (10.00)	5 (16.70)	1 (3.30)	0.092
Grade II	74 (66.07)	20 (57.14)	54 (70.13)		39 (65.00)	16 (53.3)	23 (76.70)	
Grade III	28 (25.00)	9 (25.71)	19 (24.68)		15 (25.00)	9 (30.00)	6 (20.00)	
Grade IV	2 (1.79)	0 (0)	2 (2.60)		0 (0)	0 (0)	0 (0)	
Preoperative Hemoglobin (g/L, mean ± SD)	123.41 ± 19.31	125.34 ± 23.30	122.53 ± 17.30	0.479	124.43 ± 20.73	125.33 ± 24.07	123.53 ± 17.13	0.740
Serum Total Bilirubin (umol/L, IQR)	52.75 (18.13 - 165.3)	33.2 (19.8 - 158)	56.3 (18.05 - 172.0)	0.925	34.75 (16.60 - 146.9)	32.8 (15.83 - 156.05)	39.30 (17.25 - 110.15)	0.779
Pathology [n (%)]								
Pancreatic Disease	64 (57.14)	21 (60.00)	43 (55.84)	0.622	33 (55.00)	18 (60.00)	15 (50.00)	0.337
Benign	7 (6.25)	3 (8.57)	4 (5.19)		4 (6.67)	3 (10.00)	1 (3.33)	
Neuroendocrine	3 (2.68)	2 (5.71)	1 (1.30)		2 (3.33)	2 (6.67)	0 (0)	
Malignant	46 (41.07)	14 (40.00)	32 (41.56)		21 (35.00)	11 (36.67)	10 (33.33)	
IPMN	8 (7.14)	2 (5.71)	6 (7.79)		6 (10.00)	2 (6.67)	4 (13.33)	
Ampullary Disease	10 (8.93)	2 (5.71)	8 (10.39)		6 (10.00)	1 (3.33)	5 (16.67)	
Biliary Tract Disease	15 (13.39)	3 (8.57)	12 (15.58)		5 (8.33)	2 (6.67)	3 (10.00)	
Duodenal Disease	23 (20.54)	9 (25.71)	14 (18.18)		16 (26.67)	9 (30.00)	7 (23.33)	

IQR, Interquartile range; PPPD, Pylorus preserving pancreaticoduodenectomy; DFA, Drain fluid amylase; ASA, American society of anesthesiologists; IPMN, Intraductal papillary mucinous neoplasm.

* and bold values represent statistical significance (p < 0.05).

**Table 2:** Postoperative complications of enrolled participants in retrospective cohort study.

	Original Cohort				Propensity Score Matching			
	Total(n = 112)	EDR(n = 35)	LDR(n = 77)	P_Value	Total(n = 60)	EDR(n = 30)	LDR(n = 30)	P_Value
Drain Placement Time (POD day, IQR)	11 (3 - 15.75)	3	13 (11 - 20)	< 0.000*	4.5 (3 -13.75)	3	13.5 (10.75 - 19.25)	< 0.000*
Grade B/C POPF [n (%)]	5 (4.46)	2 (5.71)	3 (3.90)	1.000	3 (5.00)	1 (3.33)	2 (6.67)	1.000
Total Complications [n (%)]	31 (27.68)	6 (17.14)	25 (32.47)	0.093	14 (23.33)	4 (13.33)	10 (33.33)	0.067
Grade 2-4 Complications [n (%)]	26 (23.21)	4 (11.43)	22 (28.57)	0.046*	10 (16.67)	2 (6.67)	8 (26.67)	0.038*
Postpancreatectomy Hemorrhage [n (%)]	4 (3.57)	0 (0)	4 (5.19)	0.307	2 (3.33)	0 (0)	2 (6.67)	0.492
Intra-abdominal Infection [n (%)]	7 (6.25)	0 (0)	7 (9.09)	0.096	3 (5.00)	0 (0)	3 (10.00)	0.237
Post-operative in-hospital Stay (day, IQR)	14 (11 - 21)	11 (9 - 14)	15 (12.5 - 22.5)	< 0.0001*	13 (10 - 16)	11 (9 - 14)	15 (11.75 - 21.5)	< 0.0001*
Delayed Gastric Emptying [n (%)]	12 (10.71)2	3 (8.57)	9 (11.69)	0.869	4 (6.67)	2 (6.67)	2 (6.67)	1.000
Biliary Fistula [n (%)]	6 (5.36)	0 (0)	6 (7.79)	0.174	3 (5.00)	0 (0)	3 (10.00)	0.237
Intra-abdominal Fluid Collections [n (%)]	7 (6.25)	3 (8.57)	4 (5.19)	0.792	4 (6.67)	2 (6.67)	2 (6.67)	1.000
Wound Infection [n (%)]	0 (0)	0 (0)	0 (0)	NA	0 (0)	0 (0)	0 (0)	NA
Pulmonary Complications [n (%)]	1 (0.89)	0 (0)	1 (1.30)	1.000	0 (0)	0 (0)	0 (0)	NA
Mortality [n (%)]	2 (1.79)	0 (0)	2 (2.60)	1.000	1 (1.67)	0 (0)	1 (3.33)	1.000
Intervention [n (%)]	3 (2.68)	0 (0)	3 (3.90)	0.551	1 (1.67)	0 (0)	1 (3.33)	1.000
Re-admission [n (%)]	5 (4.46)	3 (8.57)	2 (2.60)	0.355	2 (3.33)	2 (6.67)	0 (0)	0.492
Re-operation [n (%)]	1 (0.89)	0 (0)	1 (1.30)	1.000	0 (0)	0 (0)	0 (0)	NA

POPF, postoperative pancreatic fistula.

* and bold values represent statistical significance (p < 0.05).

### Primary and secondary outcomes in single-center study

**Table 2** described postoperative complications of enrolled participants. There were no statistical differences between EDR and LDR group in primary outcomes, including Grade B/C POPF (Original cohort: 5.71% vs. 3.90%; P = 1.000; PSM cohort: 3.33% vs. 6.67%; P = 1.000), and total complications (Original cohort: 17.14% vs. 32.47%; P = 0.093; PSM cohort: 13.33% vs. 33.33%; P = 0.067).

EDR was associated with a decrease of Grade 2-4 complications (Original cohort: 11.43% vs. 28.57%; P = 0.046; PSM cohort: 6.67% vs. 26.67%; P = 0.038), post-operative in-hospital stay (Original cohort: 11 [9 - 14] vs. 15 [12.5 – 22.5]; P < 0.0001; PSM cohort: 11 [9 - 14] vs. 15 [11.75 – 21.5]; P < 0.0001). No significant differences were observed in single abdominal complications, including PPH (Original cohort: 0 vs. 5.19%; P = 0.307; PSM cohort: 0 vs. 6.67%; P = 0.492), intra-abdominal infection (Original cohort: 0 vs. 9.09%; P = 0.096; PSM cohort: 0 vs. 10.00%; P = 0.237), delayed gastric emptying (Original cohort: 8.57 vs. 11.69%; P = 0.869; PSM cohort: 6.67 vs. 6.67%; P = 1.000), and intra-abdominal fluid collections (Original cohort: 8.57 vs. 5.19%; P = 0.792; PSM cohort: 6.67 vs. 6.67%; P = 1.000). The rates of biliary fistula, wound infection, pulmonary complications between two groups were also comparable. The mortality, intervention, re-admission re-operation occurred in 0/0/3/0 patients in EDR group versus 2/3/2/1 patients in LDR group without significant differences. After PSM, the results were the same.

### Exploring risk factors of total complications after PD

The correlation analysis of total complications and multiple characteristics were summarized in **Table 3**. Total complications were related to age, operation time, ASA scores, serum total bilirubin, and pathology. After continuous variables being converted into categorical variables, univariate logistic regression was performed, and the result showed that pathological characteristic was related to total complications. Early drain removal decreased slightly the total complications rate, but it was not significant difference (OR = 0.430; P = 0.098) (**Table 4**). Finally, the variables (P < 0.1) were included into multivariate logistic regression analysis, which also proved that only pathological characteristic was the independent risk factor associated with the incidence of total complications compared with benign pancreatic diseases (IPMN: OR = 0.087; P = 0.024; duodenal disease: OR = 0.098; P = 0.049) (**Table 4**).

**Table 3:** Correlation analysis of total complications of enrolled participants in retrospective cohort study.

	Original Cohort		
	Total Complications: Yes (n = 31)	Total Complications: No (n = 81)	P_Value
Age (year, mean ± SD)	67.39 ± 11.08	62.33 ± 12.35	0.049*
Gender [female, n (%)]	13 (41.94)	34 (41.98)	0.997
BMI (kg/m2, mean ± SD)	24.37 ± 3.53	23.31 ± 3.07	0.122
Pancreatitis [n (%)]	5 (16.13)	12 (14.81)	1.000
Diabetes [n (%)]	10 (32.26)	21 (25.93)	0.503
Cardiovascular Disease [n (%)]	5 (16.13)	14 (17.28)	0.884
Smoke [n (%)]	10 (32.26)	26 (32.10)	0.987
Alcohol [n (%)]	5 (16.13)	20 (24.69)	0.330
Neoadjuvant Chemotherapy [n (%)]	1 (3.22)	1 (1.23)	0.479
Soft Pancreatic Texture [n (%)]	11 (35.48)	31 (38.27)	0.785
Operation Time (min, IQR)	327 (271 - 413)	285 (240 - 349)	0.019*
Blood Loss (ml, IQR)	300 (150 - 500)	200 (100 - 300)	0.050
Intraoperative RBC transfusion [n (%)]	6 (19.35)	11 (13.58)	0.640
Diameter of Main Pancreatic Duct < 3 mm [n (%)]	13 (41.94)	37 (45.68)	0.721
PPPD [n (%)]	17 (54.84)	56 (69.14)	0.155
DFA on POD 1 (U/L, IQR)	478 (138 - 1532)	452 (107.5 - 1754)	0.642
DFA on POD 3 (U/L, IQR)	181 (43 - 1340)	123 (24 - 700)	0.301
Vascular Resection [n (%)]	2 (6.45)	2 (2.47)	0.655
ASA Score [n (%)]			
Grade I	0 (0)	8 (9.88)	0.022*
Grade II	19 (61.29)	55 (67.90)	
Grade III	12 (38.71)	16 (19.75)	
Grade IV	0 (0)	2 (2.47)	
Preoperative Hemoglobin (g/L, mean ± SD)	122.81 ± 19.63	123.64 ± 19.31	0.839
Serum Total Bilirubin (umol/L, IQR)	95.5 (24.3 - 239.1)	33.2 (15 - 155.4)	0.033*
Pathology [n (%)]			
Pancreatic Disease	21 (67.74)	43 (53.09)	0.048*
Benign	2 (6.45)	5 (6.17)	
Neuroendocrine	1 (3.22)	2 (2.47)	
Malignant	17 (54.84)	29 (35.80)	
IPMN	1 (3.22)	7 (8.64)	
Ampullary Disease	3 (9.68)	7 (8.64)	
Biliary Tract Disease	6 (19.35)	9 (11.11)	
Duodenal Disease	1 (3.22)	22 (27.16)	
Drain Placement Time [EDR, n (%)]	6 (19.35)	29 (35.80)	0.093

* and bold values represent statistical significance (p < 0.05).

**Table 4:** Univariate and multivariate logistic regression of total complications of enrolled participants in retrospective cohort study.

	Univariate Analysis		Multivariate Analysis	
	OR (95% CI)	P_Value	OR (95% CI)	P_Value
Age (> 65/<= 65)	0.550 (0.238 - 1.270)	0.161		
Gender (F/M)	1.002 (0.433 - 2.317)	0.997		
BMI (> median/<= median)	0.765 (0.333 - 1.755)	0.527		
Pancreatitis (N/Y)	1.106 (0.355 - 3.446)	0.862		
Diabetes (N/Y)	1.361 (0.552 - 3.354)	0.504		
Cardiovascular Disease (N/Y)	0.920 (0.301 - 2.812)	0.884		
Smoke (N/Y)	1.007 (0.415 - 2.443)	0.987		
Alcohol (N/Y)	0.587 (0.199 - 1.731)	0.334		
Neoadjuvant Chemotherapy (N/Y)	2.667 (0.162 - 44.001)	0.493		
Soft Pancreatic Texture (N/Y)	0.887 (0.375 - 2.099)	0.785		
Operation Time (> median/<= median)	0.531 (0.228 - 1.236)	0.142		
Blood Loss (> 200/<= 200)	0.495 (0.214 - 1.147)	0.101		
Intraoperative RBC transfusion (N/Y)	1.527 (0.511 - 4.563)	0.448		
Diameter of Main Pancreatic Duct < 3 mm (N/Y)	0.859 (0.372 - 1.983)	0.722		
PPPD (N/Y)	0.542 (0.232 - 1.268)	0.158		
DFA on POD 1 (> median/<= median)	0.915 (0.400 - 2.094)	0.833		
DFA on POD 3 (> median/<= median)	0.905 (0.396 - 2.194)	0.826		
Vascular Resection (N/Y)	2.724 (0.367 - 20.242)	0.327		
ASA Score (III - IV/I - II)	0.452 (0.185 - 1.104)	0.082	0.495 (0.186 - 1.321)	0.160
Preoperative Hemoglobin (> median/<= median)	0.728 (0.317 - 1.671)	0.454		
Serum Total Bilirubin (> median/<= median)	0.440 (0.187 - 1.036)	0.060	0.507 (0.178 - 1.443)	0.203
Pathology [n (%)]				
Benign	1		1	
Neuroendocrine	0.114 (0.009 - 1.514)	0.100	0.081 (0.006 – 1.159)	0.064
Malignant	0.091 (0.004 - 2.073)	0.133	0.067 (0.003 - 1.599)	0.095
IPMN	0.078 (0.010 - 0.628)	0.017*	0.087 (0.010 - 0.720)	0.024*
Ampullary Disease	0.318 (0.018 - 5.779)	0.439	0.253 (0.013 – 4.908)	0.364
Biliary Tract Disease	0.106 (0.009 - 1.190)	0.069	0.176 (0.015 -2.132)	0.172
Duodenal Disease	0.068 (0.007 - 0.650)	0.020*	0.098 (0.010 -0.994)	0.049*
Drain Placement Time (3/>3)	0.430 (0.158 - 1.170)	0.098	0.473 (0.160 - 1.398)	0.176

CI, Confidence interval.

* and bold values represent statistical significance (p < 0.05).

### Management of postoperative pancreatic fistula

In total, the rate of Grade B/C POPF was 4.46% (5/112), which occurred in 2 patients in EDR and 3 patients in LDR (P = 1.000). Low Grade B/C POPF rate indicated that low risk patient selection strategy (DFA on POD 1 and 3 <= 5000 U/L) works. EDR group had 2 grade B POPF. In contrast, LDR had 2 grade B POPF and 1 grade C POPF. Postoperative course of patients with pancreatic fistula were recorded in (**Table 5**). The patient had grade C POPF in LDR group even though DFA on POD 1 and 3 < 40 U/L, who underwent PD because of cholangiocarcinoma. Drains were removed on POD 8. Reoperation was conducted to explore for dehiscence of the anastomotic stoma and hemorrhage, which significantly extended in-hospital stay for 77 days, and finally caused mortality. Three of five patients with Grade B/C POPF had positive drain fluid cultures, thus antibiotic therapy was established according to drug sensitivity test. Percutaneous drain insertion or conservative treatment were used for grade B POPF.

**Table 5:** Characteristics of patients with Grade B/C POPF.

ID	Group	Soft PancreaticTexture	Diameter of Main PancreaticDuct < 3 mm	Blood Loss(ml)	Pathology	DFA on POD 1(U/L)	DFA on POD 3 (U/L)	Drain Placement Time (POD day)	Grading of POPF	POPF Management	Drain Fluid Culture
1	EDR	Y	Y	100	Moderately differentiated cholangiocarcinoma	3509	861	3	B	Percutaneous drain insertion	Enterococcus fecalis
2	EDR	N	N	100	Benign pancreatic disease	1133	3835	3	B	Percutaneous drain insertion	Enterococcus fecalis, Monilia albican
3	LDR	N	N	300	Moderately differentiated pancreatic ductal adenocarcinoma	463	79	26	B	Percutaneous drain insertion	Negative
4	LDR	Y	N	300	Moderately differentiated ampullary adenocarcinoma	3114	446	33	B	Conservative	Negative
5	LDR	N	N	500	Moderately differentiated cholangiocarcinoma	34	30	8	C	Postoperative hemorrhage, reoperation	Klebsiella pneumoniae

## Discussion

One of major concerns of EDR is intra-abdominal fluid collection, and caused infection and hemorrhage. This single-center study indicated that EDR could not increase the risk of intra-abdominal fluid collection and hemorrhage. The selection strategy of low risk patients: DFA on POD 1 <= 5000 U/L was utilized by previous studies (15–18, 23), and low Grade B/C POPF rate was observed in these studies. Single and multiple-center RCT performed by Dai and our single-center retrospective study used the stricter selection criteria (DFA on POD 1 and 3 <= 5000 U/L). The Grade B/C POPF rates were 1.75%, 5.13%, and 4.46% respectively. The strict selection strategy guarantees the safety of early drain removal, and aid in surgeon confidence to make a decision of EDR. However, the stricter selection strategy will narrow the clinical application of EDR. Thus, it is very important to balance selection criteria and the range of clinical application of EDR. Nowadays, the selection strategy for low risk of POPF, time-point of EDR, DFA cut-off value remains to be further investigated.

The definitions of EDR and LDR varied among previous studies. Bassi et al. (15) chose POD 3 as EDR time-point because the change of drain effluent happens on POD 3 which is also regarded as relatively early time-point, and POD 5 as LDR time-point because POD 5 is standard drain removal time-point in their institution. In our single-center study, we also chose POD 3 as EDR time-point, and >= POD 5 as LDR time-point that is the same as RCTs performed by Dai et al. (16, 17). It is better to select appropriate time-point of EDR and LDR according to local medical conditions. Our single-center study supported that EDR is safe and significantly decrease postoperative in-hospital stay (11 [9 – 14] vs. 15 [12.5 - 22.5]), indicating that faster recovery, lower medical costs for patients, in line with the idea of enhanced recovery after surgery (ERAS). In additions, Dai et al. (17) found late drain removal and laparoscopic procedure were the independent risk factors of major complications using multiple regression analysis. However, our single-center study did not prove the LDR was an independent risk factor of total complications (OR = 0.473, 95% CI: 0.160 - 1.398; P = 0.176), which might be attributed to small sample size.

Our work provides an evidence for the safety of EDR, and help promote the practice of ERAS after PD. However, there are some limitations in this study, such as (a) single-center retrospective cohort study with limited sample size; (b) relatively strict low risk patient selection criteria to narrow the clinical application range of EDR; (c) single-center study only focus on PD, the safety of EDR for DP remains to be explored.

In conclusion, our study demonstrates that early drain removal on POD 3 is safe for patients following PD with low risk of POPF.

## Data availability statement

The original contributions presented in the study are included in the article/supplementary material. Further inquiries can be directed to the corresponding authors.

## Ethics statement

The studies involving human participants were reviewed and approved by This retrospective cohort study was approved by the Ethical Committee on Peking University First Hospital (Approval No.2021-636). The ethics committee waived the requirement of written informed consent for participation.

## Author contributions

Conceptualization, KC, XDT. Literature Search, KC, XHX, ZHL. Data Collection, KC, YSM. Formal Analysis, KC. Validation, KC, XHX, FW, SPZ, ZJS, and XDT. Investigation and Visualization, KC. Methodology, KC and XDT. Writing – original draft, KC. Project administration, YMY. Writing – review & editing XDT. Supervision, XDT and YMY. All authors read and approved the final version of the manuscript.

## Funding

This study was supported by The Natural Science Foundation of China (NO.82171722, 81871954) and Beijing Municipal Natural Science Foundation (NO.7212111).

## Acknowledgments

We would like to thank all the patients enrolled at Peking University First Hospital, Qi Wang for statistical analysis.

## Conflict of interest

The authors declare that the research was conducted in the absence of any commercial or financial relationships that could be construed as a potential conflict of interest.

## Publisher’s note

All claims expressed in this article are solely those of the authors and do not necessarily represent those of their affiliated organizations, or those of the publisher, the editors and the reviewers. Any product that may be evaluated in this article, or claim that may be made by its manufacturer, is not guaranteed or endorsed by the publisher.
